# Advancements in Visual Field Testing: A Systematic Review of the 24-2C Test Grid

**DOI:** 10.3390/bioengineering12070711

**Published:** 2025-06-29

**Authors:** Eric Jin, Natalie Shi Qi Wong, Claire Xin Yi Goh, Michael W. Stewart, Syril Dorairaj, Bryan Chin Hou Ang

**Affiliations:** 1 Yong Loo Lin School of Medicine, National University of Singapore, Singapore 117597, Singapore; 2 Department of Ophthalmology, Tan Tock Seng Hospital, National Healthcare Group Eye Institute, Singapore 308433, Singapore; 3 Department of Ophthalmology, Mayo Clinic, Jacksonville, FL 32206, USA; 4 Lee Kong Chian School of Medicine, Nanyang Technological University, Singapore 308232, Singapore

**Keywords:** glaucoma, visual field, 24-2C

## Abstract

A systematic review was conducted of studies published up to 30 August 2024. Studies comparing conventional visual field (VF) indices, ability to detect central visual field defects (CVFDs), structure–function (S-F) concordance, and test characteristics across the HVF 24-2C SITA-Faster, 24-2 SITA-Standard/Faster, and 10-2 SITA-Standard/Fast tests were included. Eight studies with 1239 subjects (49.1% male; mean age, 54.8–66.9 years) were analyzed. The 24-2C produced similar global VF indices compared to the 10-2 and 24-2 (Standard/Faster) (ICC = 0.95 and 0.80, *p* < 0.001), detected more VF defects and CVFD clusters, and demonstrated greater S-F concordance than the 24-2 (Standard/Faster). Although the 10-2 (Standard/Fast) detected significantly more CVFDs and had a greater S-F concordance, the agreement between the 24-2C and 10-2 grids was substantial (ĸ = 0.488 to 0.708). The 24-2C was also faster compared to the 24-2 Standard and 10-2 (Standard/Fast), with comparable false positives, higher false negatives, and fewer fixation losses than the 24-2 Standard. In conclusion, the HVF 24-2C is quicker and identifies more CVFDs than the 24-2 grid, demonstrates high agreement with the 10-2 grid, and aids in CVFD screening.

## 1. Introduction

Glaucoma is a chronic, progressive eye condition characterized typically by elevated intraocular pressure (IOP) and progressive irreversible optic nerve damage, which can ultimately lead to vision loss if left untreated [1]. The global prevalence of glaucoma is rapidly increasing because of an aging population, greater public awareness, and increased ophthalmic screening [2]. The number of glaucoma patients aged 40–80 years is projected to grow from 76.0 million in 2020 to 111.8 million by 2040 [3], which will require streamlining of glaucoma diagnosis and management to limit total devoted time and reduce associated costs.

Visual field (VF) defects are the hallmark of perimetric glaucoma, with peripheral VF loss conventionally believed to precede central visual field loss [4]. However, it has been shown that up to 28% of early glaucoma and 64% of moderate glaucoma patients have central visual field defects (CVFDs, i.e., within the central five degrees of the visual field) [5]. Central vision is essential for many activities of daily living (e.g., reading and driving) [6], and the early detection of these CVFDs is critical for early managing of glaucoma and reducing long-term disease morbidity.

The Humphrey Visual Field (HVF) is a widely used diagnostic tool for evaluating the visual field by generating a graphical map of central, paracentral, and peripheral visual field using stimuli of varying intensities [7]. Among its various test grids, the HVF 24-2 test grid is a commonly used perimetry test for assessing and monitoring field loss in glaucoma patients. However, due to the wide distances that exist between points within the central 10 degrees of the VF, it may miss subtle CVFDs [8]. In comparison, the higher sampling density of the 10-2 test grid more sensitively detects CVFDs that may be missed by the 24-2 test grid and better monitors central VF progression [9,10]. The 10-2 test grid better detects CVFDs, but its testing area is too narrow to capture peripheral VF defects, which are also critical in glaucoma assessment.

**Figure 1:**
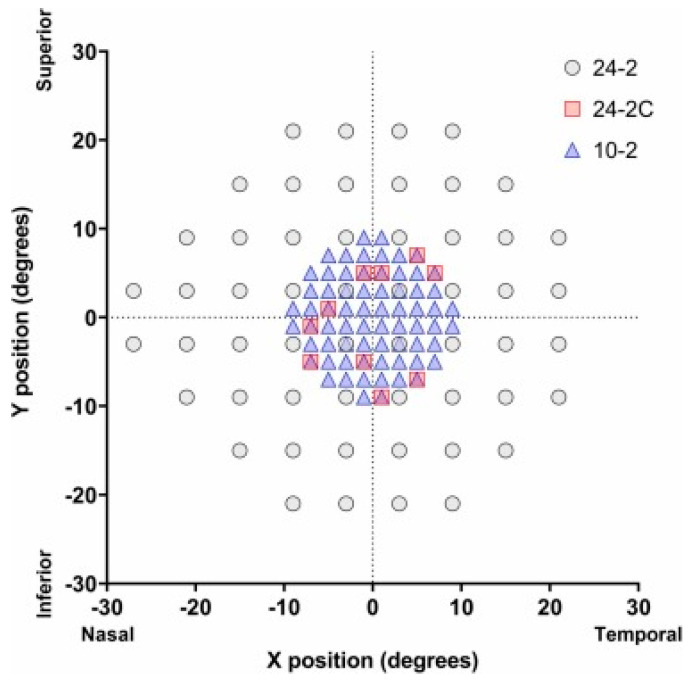
Test point locations of the 24-2C test grid compared to the 10-2 and 24-2 [16].

The newly developed 24-2C test grid addresses these limitations by integrating 10 additional VF points that are commonly affected in glaucoma within the central 10 degrees, while still using the conventional 24-2 layout (Figure 1), thereby enhancing its ability to detect CVFDs [11]. The 24-2C test grid better detects CVFDs, while shortening the testing duration and improving the structural–function (S-F) concordance compared to the 24-2 grid [11,12,13].

Previous studies have compared the HVF 24-2 and 10-2 tests grids [14,15], but this systematic review instead compares the diagnostic ability of the 24-2C test grid (conventional VF indices; ability to detect VF defects, S-F concordance, and other characteristics) with the conventional 24-2 and 10-2 test grids.

## 2. Methods

This systematic review adhered to the Preferred Reporting Items for Systematic Reviews and Meta-Analyses (PRISMA) checklist and guidelines (Supplementary Materials S1) [17]. The study protocol has been published in the International Prospective Register of Systematic Reviews (PROSPERO, CRD420251033492). Published data from the public domain was included, but since individual-level data was not used, institutional review board approval was not required. All research adhered to the tenets of the Declaration of Helsinki.

### 2.1. Search Strategy

Searches of the PubMed, EMBASE, and Cochrane Library databases from inception until 30 August 2024, were performed. Key search terms included “24-2C”, “visual field”, “perimetry”, and their synonyms. The detailed search strategy is available in Supplementary Materials S2. Reference lists of manuscripts were hand-searched for additional relevant articles. Three reviewers (EJ, CXYG, and NSQW) independently performed the literature search, with title and abstract screening, before cross-checking their lists of identified manuscripts. Differing opinions regarding suitability were adjudicated by the senior author (BCHA).

### 2.2. Eligibility Criteria

Pertinent retrospective and prospective cohort studies, cross-sectional studies, and case series were included. All English language manuscripts that included the HVF 24-2C test were included, with no restriction on the SITA mode. The following were excluded: non-English manuscripts, where the corresponding English translation of the full-text was not available; editorials, correspondences, non-human studies, letters, reviews, conference abstracts, and case reports; and studies that did not include the 24-2C.

The 24-2 grid comprises 54 test points which cover the central 24 degrees of the visual field (extending to 30 degrees nasally), and 12 of those test points lie within the central 10 degrees of fixation. In contrast, the 10-2 grid includes 68 test points concentrated entirely within the central 10-degrees region [7]. The 24-2C grid, meanwhile, builds upon the 24-2 by incorporating 10 additional asymmetrically distributed points within the central 10 degrees [18].

### 2.3. Data Collection and Risk-of-Bias Assessment

The following information was manually extracted from the full texts of included studies: demographics and patient conditions; type of HVF grid included (24-2C, 24-2, and 10-2); comparative results between the 24-2C and 24-2 grids; comparative results between the 24-2C and 10-2 grids; and other clinical utilities of the 24-2C test.

Three reviewers (EJ, CXYG, and NSQW) used a pre-defined template to independently extract information and compare the results to ensure accuracy in data collection. The risk of bias of included studies was independently assessed by three reviewers (EJ, CXYG, and NSQW) using the Joanna Briggs Institute (JBI) checklist for analytical cross-sectional studies. Each question has three options of “yes”, “no”, or “unclear”, and these account for 1 point, 0 point, and 0.5 point, respectively. Studies were considered to have a high risk of bias if the total score was less than 3 points, moderate risk of bias and moderate quality if the total score was 3–5.5 points, and low risk of bias if the total score was 6–8 points [19]. Differing opinions were adjudicated by the senior author (BCHA).

### 2.4. Data Synthesis and Analysis

The 24-2C grid was compared with the 24-2 and 10-2 grids based on the following outcomes: ability to detect VFD and CVFDs; comparison of conventional VF indices; structural–functional correlations; and test characteristics, such as reliability indices and test duration. Due to the high heterogeneity among included studies in terms of test parameters, SITA modes, and outcome definitions, direct statistical pooling (meta-analysis) was not feasible, and data was qualitatively synthesized instead.

## 3. Results

### 3.1. Summary of Included Studies

**Figure 2:**
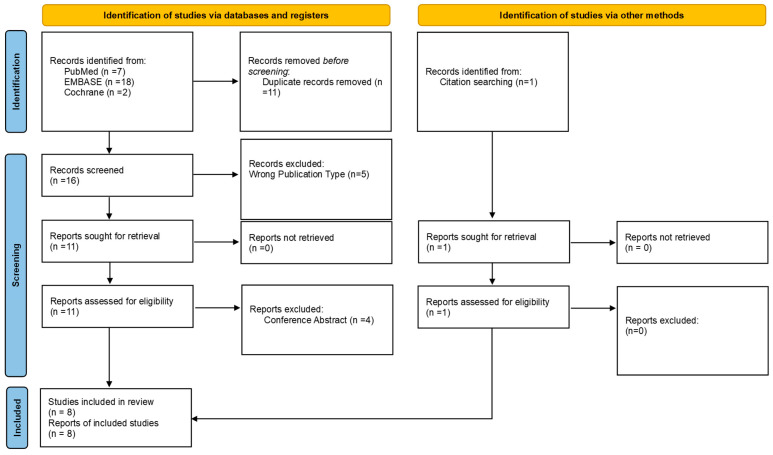
PRISMA flowchart.

A total of eight studies with 1239 subjects (596/1214 males; 49.1%) were included (Figure 2); one study did not report the gender distribution of its participants [20]. The mean ages ranged from 54.8 [20] to 66.9 [13] years (Table 1). Most of the studies (6/8; 75.0%) included only glaucoma patients or glaucoma suspects, while one study included neuro-ophthalmology patients [20], and another included all patients scheduled for VF tests without providing specific indications for this testing [21]. Three studies were from North America [13,20,21], three from Asia [12,22,23], and two from Oceania [11,18].

Two studies compared the 24-2C and 24-2 grids, three studies compared the 10-2 and 24-2C grids, two studies compared all three grids, and one study examined only the 24-2C grid. All 24-2C grids used the Swedish Interactive Threshold Algorithm—Faster (SITA-Faster) mode, while both the 24-2 and 10-2 grids used the SITA-Standard and SITA-Faster modes in four [11,13,22,23] and three studies [11,12,21], respectively.

A CVFD was diagnosed using the cluster criteria, which required a cluster of three contiguous abnormal points with any of the following conditions: two points with *p* < 0.05 and one point with *p* < 0.01; one point each with *p* < 0.05, *p* < 0.02, and *p* = 0.02; at least three points at the *p* < 0.05 level, where at least one point is at the *p* < 0.01 level; or a pair of contiguous points at the *p* < 0.01 level, within a hemifield on either the total deviation (TD) or pattern deviation (PD) plot. One study required corroboration of the cluster criteria with defects on ganglion cell analysis (GCA) [23]. CVFDs were defined as VFDs within the central 10 degrees (five out of eight studies) or 20 degrees (one out of eight studies) [18], while the remaining two studies did not explore CVFDs. Notably, three studies [20,21,22] did not provide a definition for VFDs.

### 3.2. Risk-of-Bias Assessment

All studies had a low risk of bias, but two studies [20,21] lacked sufficient information regarding the selection criteria for visual field test results and patient conditions (Supplementary Materials S3).

### 3.3. Summary of Findings

A summary of the comparisons between the three grids is provided in Table 2, while detailed comparisons between the 24-2C and the 24-2 and 10-2 grids are presented in Table 3, Table 4, Table 5 and Table 6.

*(1)*  *Comparison of 24-2C and 24-2 test grids*

**Detecting Visual Field Defects**

Three studies [11,22,23] investigated the ability of the 24-2C and 24-2 (SITA Faster and Standard) modes to detect VFDs and CVFDs; two studies [22,23] compared the 24-2C with the 24-2 Standard, while the third [11] compared the 24-2C and 24-2 on both Standard and Faster modes. Overall, the results from these studies were mixed.

Behera et al. [22] found that the 24-2C was able to detect more defective points than the 24-2 Standard in both the TD plot (17.5 vs. 12; *p* < 0.001) and the PD plot (6 vs. 4; *p* < 0.001) in the central 10° of vision. Beyond the central 10° of vision, however, there were no significant differences between the 24-2-Standard and 24-2C fields in creating TD and PD plots. Similarly, Nishijima et al. [23] found that the 24-2C (with 22 points within the central 10°) detected more CVFDs than did the 24-2 Standard (with 12 points within the central 10°) in patients with mild glaucoma in the central upper region of the total probability (TP) plot (AUC = 0.85, 95% CI = 0.78, 0.91 vs. AUC = 0.75 95% CI = 0.67, 0.83; *p* < 0.05) and the central lower region of the PD plot (AUC = 0.81, 95% CI = 0.72, 0.90 vs. AUC = 0.64, 95% CI = 0.53, 0.75; *p* < 0.05). However, both programs were similar regarding detection of CVFD in the central lower region of the TP plot and the central upper region of the PD plot.

In comparison, Phu et al. [11] demonstrated that while the 24-2C tended to identify more clusters of CVFD than the 24-2 Standard and Faster, this difference was not statistically significant across all criteria (1+ solitary, 2+ contiguous or 3+ contiguous points of reduction). When directly comparing CVFDs, however, the 24-2C successfully identified more clusters in 2 of 64 cases (3.1%) that were not found by the 24-2-Faster test.

**Conventional visual field indices**

Two studies [11,22] that directly compared conventional visual field indices (mean deviation (MD), pattern standard deviation (PSD), and central mean density (CMD)) between the 24-2C and 24-2 grids (SITA Standard and Faster) found that conventional visual field indices were comparable between the two tests.

Behera et al. [22] compared the 24-2 Standard with the 24-2C test grid in 60 glaucoma patients. The 24-2 Standard measured a lower MD (−6.9 vs. −5.5 dB; *p* = 0.01), with a mean difference of 0.891 dB (95% LOA = (−5.125 to 3.344 dB)), but it produced a higher PSD compared to the 24-2C (5.5 vs. 5.1 dB; *p* < 0.001), with a mean difference of 0.694 dB (95% LOA = (−2.409, 3.797 dB)). The Bland–Altman plot, however, showed no differences between the two test grids. Comparison of intraclass correlation coefficients (ICCs) also found that the 24-2 Standard and 24-2C were similar in terms of MD (ICC = 0.95, CI 0.92–0.97, *p* < 0.001) and PSD (ICC = 0.93, CI 0.89–0.96, *p* < 0.001). In contrast, Phu et al. [11] found that, in 104 glaucoma or glaucoma-suspect patients, the 24-2C produced a lower MD (mean difference = −0.73 dB, 96.2% CI = (−1.01 to −0.06 dB); *p* = 0.0038) compared to the 24-2-Standard test. There were no significant differences in MD between both grids when using the SITA Faster (median difference = −0.02 dB; 96.7% CI = (−0.33 to −0.30 dB), *p* = 0.9715). CMD values followed a similar pattern, with the 24-2C producing a lower CMD than the 24-2 Standard (median difference = −0.35 dB; 96.2% CI = (−0.70 to −0.03 dB), *p* = 0.0226) and no significant difference with the 24-2 Faster (median difference = 0.13 dB; 96.7% CI = (0.27, 0.04 dB); *p* = 0.2769). PSD was also not significantly different between the 24-2C and 24-2 test grids on both the Standard and Faster modes (*p* > 0.05).

**Macular S-F concordance**

Two studies evaluated the macular S-F concordance using the 24-2C and 24-2-Faster tests and both found that the 24-2C had a higher S-F concordance compared to the 24-2 Faster.

Structural–functional correlation refers to the ability of the visual field tests to detect overlapping structural and functional losses. Structural losses were defined as reduced ganglion cell–inner plexiform layer (GCIPL) thickness values on Cirrus OCT (Carl Zeiss Meditec) of the macula; statistical significance was defined at the *p* < 0.05 or *p* < 0.01 levels. The VF test locations, after adjusting for the relative displacement of Henle’s fibers, were then superimposed onto the macular OCT results to detect structural–functional concordance in one study [11], while a second superimposed the visual field mean sensitivity (VFMS) of each test point to the corresponding parafoveal GC-IPL value according to the Garway–Heath map [12].

To derive the S-F correlation, Hong et al. [12] measured the central visual field MS in six parafoveal sectors (superotemporal, superocentral, superonasal, inferotemporal, inferocentral, and inferonasal) and performed a correlation analysis to macular GCIPL (mGCIPL) thickness. Phu et al. [11] superimposed pattern deviation maps (to account for variations in age) to the ganglion cell analysis deviation maps and classified test points into four outcomes: “neither” (signifying neither a reduction in structure nor function); “both”, “structure” only; or “function” only. Hong et al. [12] found significantly stronger S-F associations in the average, hemimacular, and superotemporal and inferotemporal parafoveal sectors in the 24-2C compared to the 24-2-Faster test among all recruited patients (healthy/pre-perimetric/perimetric glaucoma patients, n = 150; *p* < 0.05) and those with perimetric glaucoma (n = 82; *p* < 0.05). The S-F correlations were similar, however, between the two test grids when these associations were compared in the superocentral + superonasal and inferocentral + inferonasal parafoveal sectors. Similarly, Phu et al. [11] found that the 24-2C identified more significant defects in regions with structural loss than the 24-2-Faster grid at both *p* < 0.05 and *p* < 0.01 significance. The 24-2C also found a significantly higher ratio of functional defects compared to structural defects compared to the 24-2 in both the 24-2-Standard and 24-2-Faster cohorts (*p* < 0.001).

**Test characteristics**

Time Taken

Three studies evaluated the testing time required for completion of the 24-2C and 24-2 test grids. Phu et al. [11] (24-2C median = 155.0 s, IQR = (140.0–174.0 s) vs. 24-2-Standard median = 314.0 s, IQR = (283.8–338.0 s); *p* < 0.001), Behera et al. [22] (24-2C median = 210.5 s, IQR = (176.25–247.25 s) vs. 24-2-Standard median = 408 s, IQR = (338.75–471.5 s); *p* < 0.001), and Nishijima et al. [23] (24-2C mean = 165.0 s, SD = (161–170 s) vs. 24-2-Standard mean = 308.0 s, SD = (302–315 s), *p* < 0.001) all found that that the 24-2C took less time to complete compared to the 24-2 Standard. The 24-2 Faster, however, took less time than the 24-2C (24-2-Faster median = 125.5 s, IQR = (110.5–148.8 s) vs. 24-2C median = 154.5 s, IQR = (136.3–181.8 s); median difference = 26.0 s, *p* < 0.0001) [11] by approximately half a minute.

Reliability Indices

One study evaluated reliability indices from the 24-2C and 24-2 Standard. Behera et al. [22] found no significant differences in false-positive rates (24-2 Standard, median = 1%, IQR = (0.0–2.8%); 24-2C (median = 0%, IQR = (0–4%); *p* = 0.34) but the 24-2C had a significantly higher false-negative rate (median = 7.5%, IQR = (0.0–12.75%) vs. median = 4.5%, IQR = (0–7%), *p* < 0.01). Fixation losses were highest with the 24-2 Standard (median = 5.5%, IQR = (0.0–12.7%)) compared to the 24-2C and 10-2 Standard.

*(2)*  *Comparison of 24-2C and 10-2 test grids*

**Ability to detect VFD**

Four studies [13,18,20,22] with 365 patients compared the 24-2C with the 10-2 visual field test (SITA Standard and Fast) to detect VFDs and CVFDs. Regarding VFD detection, Yamane et al. [20] found no significant difference between the total number of flagged points in the 10-2-Fast and 24-2C tests as measured on the TD plots at *p* < 0.05 (24-2C vs. 10-2; 4.16 (0.71) vs. 4.28 (0.73), *p* = 0.767), *p* < 0.02 (24-2 vs. 10-2; 3.15 (0.69) vs. 3.28 (0.69), *p* = 0.791) and *p* < 0.01 (24-2C vs. 10-2; 2.45 (0.63) vs. 2.58 (0.63), *p* = 0.651) in patients with neuro-ophthalmology conditions. Similarly, there were no differences in the number of flagged points on the PD plots at all three levels of significance (*p* = 0.70).

**CVFD detection**

When specifically assessing CVFD detection, three studies [13,18,22] demonstrated that the 10-2 test grid (Standard and Fast) consistently outperformed the 24-2C. Behera et al. [22] found that the 10-2 Standard detected 2.6 times more defective points (46 vs. 17.5) in the TD plot and 2.8 times more CVFDs (17 vs. 6) in the PD plot compared to the 24-2C within the central visual field. Similarly, Chakravarti et al. [13] found more CVFDs in glaucoma and glaucoma suspect eyes with the 10-2 Standard, based on both TD (58 (43.9%) vs. 42 (31.8%)) and PD plots (56 (42.4%) vs. 42 (31.8%)). The largest number of hemifield-specific CVFDs was also found on the superior 10-2-Standard TD plots (n = 45) and more CVFDs were found on the 10-2-Standard TD plots (n = 58).

Phu et al. [18] further substantiated these findings by demonstrating that the 10-2-Fast test detected significantly more CVFDs in both glaucoma patients (*p* = 0.006) and combined glaucoma patients and suspects (*p* < 0.0001), compared to the 24-2C. The 10-2 Fast also found more CVFDs (*p* = 0.02) compared to the 24-2C in glaucoma patients and suspects, though this difference was not significant when analyzing glaucoma patients alone (*p* = 0.051). The 10-2 Fast also found significantly more CVFDs compared with the 24-2C for all contiguity conditions (2+ to 6+; *p* < 0.001).

Despite the superior detection capabilities of the 10-2-Fast test, Yamane et al. [20] noted that the additional VFDs detected did not necessarily change the clinical diagnosis, since the extra VFDs were deemed clinically unhelpful in nine of 25 cases. Moreover, there was strong agreement between the VFDs detected by both test grids. Chakravarti et al. [13] found moderate-to-substantial agreement (ĸ = 0.488 to 0.708) for identifying superior, inferior, or any CVFD between 10-2-Standard and 24-2C test grids on TD and PD plots, with agreement highest for detecting any superior CVFDs (ĸ = 0.708) and lowest for detecting inferior arcuate and partial arcuate CVFDs (ĸ = 0.477) on TD plots. For PD plots, agreement was best for detecting any inferior CVFDs (ĸ = 0.689) and worst for detecting superior arcuate and partial arcuate CVFDs (ĸ = 0.397) [13].

**Comparison of conventional visual field indices**

Two studies [18,22] with 225 patients directly compared conventional visual field indices between the 24-2C and 10-2 test grids (SITA Standard and Fast), with both studies showing that the MD and PSD were similar between both test grids.

Behera et al. [22] found that the 24-2C produced smaller MD results compared to 10-2 Standard (Mean difference = 1.635 dB, 95% LOA = (−9.124 to 5.854 dB)), while delivering greater PSD results (Mean difference = 0.776 dB, 95% LOA = (−4.678 to 6.230 dB)). The Bland–Altman plots, however, showed no systematic differences between the two tests. ICC also showed that both tests produced similar MD (ICC = 0.80, 95% CI = (0.687, 0.876), *p* < 0.001) and PSD (ICC = 0.80, 95% CI = (0.690–0.877), *p* < 0.001) results. Phu et al. [18] similarly found minimal differences in the MD (Mean bias = 0.70 dB, 95% LOA = (−3.8 to 5.2 dB)) and PSD (Mean bias = 0.92 dB, 95% LOA = (−5.0 to 3.2 dB)) results as depicted by small bias on Bland–Altman analyses between the 24-2C and 10-2 Fast. CMD was also similar between both visual field grids (Mean bias = 0.02 dB, 95% LOA = (−6.6 to 6.6 dB)) [18]. Phu et al. [18] also found a statistically significant positive correlation between the MD (R2 = 0.56, *p* < 0.001), PSD (R2 = 0.58, *p* < 0.001) and CMD (R2 = 0.78, *p* = 0.001) of 24-2C and 10-2-Fast tests.

**Macular S-F concordance**

Phu et al. [18] assessed macular S-F concordance between 24-2C and 10-2 Fast in 188 patients. After correcting for relative displacement of Henle’s fibers, visual field test locations were superimposed upon macular OCT scans of the GCIPL thickness values from the Ganglion Cell Analysis printout of the Cirrus OCT. Since not all visual field test locations were within this area, S-F correlation was performed with the following two conditions: where visual field test locations were superimposed on the structural scan (the “structure grid” condition); and separately at locations across all visual field test locations within the central 20 degrees (the “function grid” condition). The defects were classified as “neither” (no structural or functional loss), “both” (structural and functional loss present), “structure only,” or “function only.” The 10-2 Fast was found to produce greater structural–functional concordance, as well as more functional defects among the structural defects for both conditions (*p* < 0.0001).

**Test characteristics**

Testing Time

Three studies found that both the 10-2 Standard and Fast required a longer testing duration compared to the 24-2C. Both Nishijima et al. [23] and Behera et al. [22] found that the 10-2 Standard took a longer time to complete than the 24-2C test (24-2C mean = 165.0 s, SD = (161–170 s) vs. 10-2-Standard mean = 343 s, SD = (334–353 s), *p* < 0.001) (24-2C median = 210.5 s, 10-2-Standard median = 244.5 s). Yamane et al. [20] also showed that the 10-2 Fast took longer than the 24-2C Faster to complete (24-2C average = 3:09 min vs. 10-2-Fast average = 3:58, *p* < 0.001).

Reliability Indices

Two studies compared the reliability indices derived from the grids of the 24-2C and 10-2 in both Fast and Standard modes. Yamane et al. [20] found that the 10-2 Fast had significantly lower false-positive (1.09% vs. 2.57%, *p* = 0.043) and false-negative (1.15% vs. 3.89%, *p* = 0.002) rates compared to the 24-2C. Chakravarti et al. [13] similarly found that the 10-2 Standard had a higher specificity (1.0 vs. 0.94) and sensitivity (1.0 vs. 0.97) compared to the 24-2C.

*(3)*  *Others*

Meshkin et al. [21] investigated the feasibility of conducting simultaneous remote visual field tests using the 24-2C grid, as opposed to performing one remote visual field test at any one time. Among 861 eyes scheduled for visual field tests, no significant differences were observed between single and simultaneous testing across multiple parameters, including false-positive rates (*p* = 0.10), MD (*p* = 0.42), PSD (*p* = 0.31), VFI (*p* = 0.45), and test duration (*p* = 0.70).

## 4. Discussion

### 4.1. History of 24-2C

Functional assessment in glaucoma has traditionally been performed using two main visual field grids: the 24-2, which primarily evaluates the peripheral visual field, is used for monitoring pre-perimetric and early glaucoma; and the 10-2 test, which concentrates on the central visual field, is used for patients with advanced peripheral loss that also affects central vision. Due to time and resource constraints, both tests are not routinely performed at all clinical visits on all glaucoma patients. As a result, CVFDs may be missed when performing only 24-2 grid testing [8].

The 24-2C test grid was created for the Humphrey Field Analyzer (Carl Zeiss Meditec, Dublin, CA, USA) to address this limitation, by simultaneously assessing both peripheral and central visual fields. It incorporates the 10 most commonly abnormal central test points from the 10-2 grid into the Standard 24-2 pattern [14], with five additional points in each hemifield that are not symmetrically distributed across the vertical or horizontal midlines. Additionally, the 24-2C employs the SITA-Faster mode by default and uses the following modifications to reduce test duration compared to SITA-Standard and SITA-Fast modes: performance of only one test at perimetrically blind points, having no false-negative catch trails, use of a gaze tracker, and eliminating extra delay times [24]. The 24-2C also has fewer test locations than the 10-2 (62 vs. 68), allowing for faster test completion. Improved efficiency enhances its utility as a rapid screening tool, reduces patient fatigue, and improves test reliability [25]. Consolidating peripheral and central testing in pre-perimetric or early glaucoma means that the 24-2C test grid could reduce costs, improve access to care, and encourage adherence by reducing the number of tests per visit, all of which are important for optimal disease management [26].

Removing reliability checks, such as false-negative and blind-spot catch trials and second checks at perimetrically blind points, which in the 24-2C SITA Faster are important for detection of early, mild glaucomatous defects and their progression, raises concerns about the program’s accuracy and reliability [14,27]. Therefore, for the 24-2C Faster to replace the 24-2 and 10-2 in glaucoma screening and monitoring, it must demonstrate comparable or superior performance in key metrics, such as the ability to detect VFDs and CVFDs, conventional global indices, S-F concordance, and reliability indices.

### 4.2. Comparing the Capabilities of the 24-2C with the 24-2 and 10-2 Grids

#### 4.2.1. Detection of VFDs and CVFDs

CVFDs are common in early glaucoma and correlate with early macular damage and reduced mGCIPL thickness [14]. The American Academy of Ophthalmology has highlighted that CVFDs “may be underappreciated in the evaluation of early glaucoma” and are “essential in the diagnosis and management of glaucoma even at the earliest stages” [14].

Central vision is essential for daily functions such as stereoacuity [28], navigation, and obstacle avoidance [29], and impaired central vision increases susceptibility to falls, restricts mobility, and worsens vision-related quality of life [28,30]. Furthermore, CVFD-related visual challenges can lead to psychological distress, such as depression and anxiety [31,32]. Detecting and preventing the progression of CVFDs through early treatment, therefore, is vital for preserving the quality of life in glaucoma patients. Central vision field loss tends to progress rapidly, with over 10% of patients experiencing rapid MD loss in the central 10 degrees, which necessitates close monitoring to guide timely treatment adjustments and prevent further vision loss [33].

Our results show that the 24-2C tends to identify more CVFDs compared to the 24-2 Standard and Faster, whereas detecting visual field defects outside of the central 10 degrees was comparable between the 24-2C and 24-2 Standard. The additional 10 asymmetrically distributed central visual field test points imbedded within the 10-2 area of the 24-2C, which target areas vulnerable to glaucomatous damage, likely increase sensitivity to CVFDs [34], and the SITA-Faster mode does not appear to significantly compromise the sensitivity of detecting peripheral visual fields compared to the SITA Standard mode in 24-2 testing [22]. As such, the 24-2C may be a more effective tool for screening and monitoring both CVFDs and peripheral visual field damage compared to the 24-2 Standard, especially in peri-perimetric and early glaucoma patients with subtle CVFDs that may be missed by the more commonly used 24-2 Standard [10].

The 24-2C identifies more CVFDs, but this may not always correlate with clinical significance. Behera et al. [22] and Nishijima et al. [23] found that significantly more CVFDs were detected with the 24-2C than the 24-2 Standard, but Phu et al. [11] found that the higher number of CVFDs detected on the 24-2C compared to the 24-2 Standard and Faster were no longer significant when more stringent criteria were used to determine clinical significance. These criteria, however, were originally designed to detect larger VFDs on the 24-2 or CVFDs on the 10-2, but are not well-suited for the 24-2C, especially when detecting small CVFDs. This is because the central test points on the 24-2C are spaced further apart than those on the 10-2, thus making it harder to meet the stricter thresholds for clinical significance when multiple contiguous points of VFDs are needed. Higher-powered studies with validated test criteria designed for the central points of the 24-2C may be needed to evaluate its effectiveness in detecting clinically significant CVFDs.

Our results also suggest that the asymmetrical distribution of the 10 central points in the 24-2C grid may limit its sensitivity in specific areas. Nishijima et al. [23] found that both the 24-2C and 24-2 Standard detected a similar number of CVFDs in the central (nasal) upper region of the PD plot in patients with mild glaucoma. This may be attributed to the 24-2C’s additional test points being more temporally distributed and concentrated in the lower field, within the central 10 degrees [23].

Our results show that the 10-2 may better detect CVFDs and contiguous clusters compared to the 24-2C Faster. This is not unexpected since 24-2C sampling of the central locations is sparser than with the 10-2 grid—the 24-2C only has 22 central points in the central 10 degrees, compared to the 68 central points of the 10-2 grid [18]. The 24-2C, therefore, offers a balanced approach for pre-perimetric, early, and moderate glaucoma cases by assessing both central and peripheral vision. In contrast, the 10-2 test grid’s denser central sampling allows it to provide a more detailed characterization of central scotomas, making it useful to comprehensively characterize and trend central visual field defect progression in glaucoma patients with central visual field loss; the 10-2 is the investigation of choice for patients with advanced glaucoma with little residual peripheral vision [35,36]. Another use of the 10-2 grid is characterizing visual field defect patterns in different glaucoma subtypes, thereby helping to guide clinical management. As an example, studies show that early paracentral scotomas are more strongly associated with normal tension glaucoma than with POAG [37].

Our results show that the 10-2 detects more CVFDs but there is a moderate-to-substantial agreement between the 24-2C and 10-2 grids for detecting any CVFDs [13]. This suggests that the 24-2C is a viable option to initially evaluate both the central and peripheral visual fields in glaucoma patients by testing both central and peripheral fields in a single session, which may inform clinicians of the need for a separate 10-2 evaluation if there are suggestions of CVFDs [22]. From a health economics perspective, this saves time, expense, and personnel that would be required for routine 10-2 testing in conjunction with 24-2 testing [14].

#### 4.2.2. Global Indices

Global indices such as the MD and PSD are routinely used by clinicians to monitor glaucoma progression. MD represents the average difference from the mean of age-adjusted normal values for all tested visual field locations, while PSD measures irregularities in the visual field by averaging the difference between each threshold sensitivity value and the overall mean sensitivity. Clinicians often rely on trending global indices to monitor glaucoma progression, especially in mild-to-moderate glaucoma, with progression often defined by a statistically significant negative slope (*p* < 0.05) [38]. Ensuring similar MD and PSD values across visual field grids, therefore, would allow clinicians to seamlessly track disease progression, even if patients previously used different test grids, thereby facilitating long-term monitoring of visual field indices.

Our results show that global indices such as MD, PSD, and CMD were similar between 24-2C and 24-2 (Standard and Faster), and although two studies [11,22] reported that the 24-2 Standard had a slightly different MD and PSD compared to the 24-2C, the differences ranged from 0.694 dB to 0.891 dB and were not clinically significant. Furthermore, Bland–Altman plots and interclass correlations showed no systematic differences in the results of both grids, indicating that the 24-2C test grid provides comparable quantitative measurements [11]. This suggests that the 24-2C and 24-2 Standard/Faster may be interchangeable for detecting overall progression, thereby giving clinicians the option of the 24-2C at subsequent follow-ups for patients previously monitored with the 24-2 Standard/Faster if more sensitive evaluations for CVFDs are required.

Our results also show that global indices such as PSD and MD were similar between the 24-2C and 10-2 (Standard and Fast) grids. Behera et al. [22] found that the 24-2C produced lower MD and higher PSD results compared to 10-2 Standard, but the ICC test showed good consistency between the two grids [22]. Variability in MD and PSD results is to be expected, especially when peripheral locations are included, since patients performing multiple visual field tests may have fluctuations at test points across different visual field tests, especially at peripheral points [18,39,40]. No studies have directly compared the 24-2C and 10-2 using identical SITA modes, so observed differences may be due to different SITA modes that may produce different MD and PSD results [27].

#### 4.2.3. Application in Neuro-Ophthalmology Patients

Our results show that the 10-2-Standard and 24-2C test grids have comparable diagnostic value in neuro-ophthalmology patients by detecting similar numbers of CVFDs. Additional CVFDs identified by either grid may not ultimately affect clinical diagnosis and management, however, given that most neuro-ophthalmology conditions, such as optic neuritis, compressive optic neuropathies, or ischemic/ hemorrhagic stroke, present with extensive VFDs that may even be apparent on clinical examination, with little need for detailed HVF VFD delineation [41]. In contrast, conditions such as glaucoma with more diffuse defects that progress sub-clinically [42] may benefit more from the additional central test points. The 24-2C test grid, which serves as a hybrid between the 24-2 and 10-2, may still be clinically relevant for conditions with diffuse visual defects that require both central and peripheral field assessments (e.g., ischemic optic neuropathy and optic neuritis) [20,43].

#### 4.2.4. Macular S-F Concordance

Our results show that the 24-2C produced superior macular S-F concordance compared to the 24-2 Faster. This may be due to the wider spacing of the 24-2’s points in the central 10 degrees of vision, which might fall outside the location of retinal ganglion cells in the macula [12].

Despite this, Hong et al. [12] found that both test grids produced a statistically similar macular S-F relationship in certain macular sectors (SC + SN and IC + IN mGCIPLT sectors) possibly because fewer test points were added to the 24-2C grid in these areas [44]. However, the clinical impact of macular S-F concordance in these sectors may not be significant since studies have shown that the central and nasal parafoveal sectors are less frequently affected than the temporal parafoveal sector in glaucoma [45].

As expected, because of the increased number of central test points, the 10-2 produced higher levels of macular S-F concordance compared to the 24-2C, though the difference was relatively small. The 10-2 test grid has four times more test points within the central 20 degrees, but it only detected an additional 22% and 31% more visual field defects with and without corresponding structural deficits, respectively [18]. This might be because the additional points of the 10-2 mostly filled up pre-existing scotomas that had already been identified by the 24-2C instead of identifying new CFVDs [18]. Further studies are needed to explore the clinical and functional implications of these additional mild CVFDs detected by the 10-2 test grid and its impact on visual tasks, such as color perception or balance [18].

With greater macular S-F concordance, the 10-2 test grid may still be preferable for enhancing our understanding of the pathophysiology of CVFDs and its association with macular ganglion cell death in glaucoma. Techniques such as macular OCT angiography could be used to monitor the blood supply and health of these cells, offering a more targeted approach to managing CVFDs in glaucoma [46].

#### 4.2.5. Reliability Indices

Both the 24-2C and 24-2 Standard performed similarly with regard to reliability indices such as false-positive rates and fixation losses, with both test grids producing highly reliable results. Although the 24-2C produced a slightly higher false-negative rate, the difference may not be clinically significant, with the mean false-negative rate remaining well within the acceptable threshold of less than 33% [47]. While the 24-2C grid has slightly higher false-positive and -negative rates in detecting VFDs compared to the 10-2 (Fast and Standard), the rates remain relatively low. As reliability indices are patient-dependent [48], the 24-2C may theoretically produce less reliable results in patients with decreased attention or fixation reliability, with the removal of false-negative and blind-spot catch trials [24], although this limitation may be mitigated to some extent with gaze tracking. Our results, however, may not accurately reflect loss in sensitivity and specificity in the real-world setting since our included studies mostly excluded unreliable results. The lack of blind spots and false-negative catch trials may also render the 24-2C less accurate and reliable in patients with severe glaucoma due to the increased rate of false positives [47]. On the other hand, the faster test speed of the 24-2C Faster may translate to decreased test fatigue, as well as fewer attention and fixation losses over time.

Overall, the 24-2C remains a reliable alternative to the other test grids for detecting or ruling out VFDs. In addition, its comparable reliability indices with longer established testing protocols may allow for the same reliability criteria previously recommended by Humphrey Instruments, Inc. (San Leandro, CA, USA)—less than 20% fixation losses, less than 33% false-negative error, and less than 33% false-positive errors [49] to be applied for 24-4C grid as well.

In summary, the clinical utility of the 24-2C lies in its ability to possibly offer a single efficient and reliable test that captures both central and peripheral visual field changes, which reduces patient fatigue, facilitates earlier detection of CVFDs in early glaucoma, and minimizes the need for multiple tests.

### 4.3. Other Clinical Utilities and Implications

In one study, remote monitoring of two patients simultaneously using the 24-2C produced performance and reliability metrics comparable to those observed when monitoring a single patient, thus suggesting that simultaneous remote testing does not compromise accuracy [21]. Busy clinical practices may, therefore, consider reducing costs and improving efficiency by having one technician supervise multiple tests simultaneously [50]. The ability of patients to be tested simultaneously in different rooms also mitigates the lapses in concentration brought about by same-room simultaneous testing [50] and offers social-distancing benefits during pandemics. However, a greater proportion of simultaneous tests compared to single tests required restarting or conversion to in-person tests, possibly due technician unfamiliarity with the relatively novel 24-2C [21].

### 4.4. Limitations

Our review has its limitations. Firstly, the heterogeneity in experimental design and reporting methods prevented a meta-analysis, thereby limiting our ability to quantitatively assess various performance characteristics of the 24-2C test grid. Secondly, no longitudinal studies to date have explored the utility of the 24-2C grid in monitoring visual field progression compared to the 24-2 and 10-2 test grids, so the long-term utility of the 24-2C grid alone for disease staging and treatment titration remains unclear [11]. Thirdly, differences in test modes (SITA Standard, Fast, and Faster) across studies might have affected visual field results [51]—the SITA Fast and Faster are designed to reduce testing duration, but they utilize different threshold sensitivities compared to the SITA Standard [24] that have been shown to result in better visual field results and fewer significantly depressed test points [24,52]. While these differences are generally minimal, not statistically significant, and unlikely to impact our findings, they warrant further investigation [24]. Fourthly, the results reported should also be interpreted with caution because of the limited number of included studies (n = 8) due the relatively recent emergence of the 24-2C. Some comparisons of individual outcome parameters were also based on findings from only one or two studies, which may introduce a potential interpretation bias. Lastly, consecutive testing in the same patient, as performed in some studies [11,22], may introduce bias due to patient fatigue, thereby resulting in reductions in the MD and other global indices [53]. Nonetheless, the exclusion of unreliable test results (e.g., those with high fixation losses or false positives/negatives) in most studies likely mitigates this concern.

## 5. Conclusions and Future Directions

The 24-2C test grid represents a valuable alternative for assessing both the peripheral and central visual fields in patients and could replace the 24-2 to screen for and follow CVFDs in early glaucoma. Its ability to balance efficiency and reliability is particularly relevant given the increasing demand for visual field testing and the growing recognition of CVFDs in early glaucoma. The 10-2 grid remains superior, however, for detecting and characterizing CVFDs and should still be used to follow central visual losses found on an initial 24-2C grid, or in advanced glaucoma patients. As such, while the 24-2C test grid does not fully replace the detailed central assessment of the 10-2 grid, it may serve as a practical and efficient first-line tool that balances central and peripheral visual field testing in glaucoma patients with pre-perimetric or early disease. Beyond glaucoma, the 24-2C test grid may be useful for neuro-ophthalmology patients or for conditions that require both central and peripheral visual field monitoring. Additional prospective comparative studies comparing the performance characteristics and clinical utility of all three visual field test grids will be helpful to determine the optimal strategy for disease detection and monitoring in all stages of glaucoma.

## Figures and Tables

**Table 1:** Study characteristics.

S/N	Author	Year	Country (City)	Number of Participants (M/F)	Age, Mean (SD)	Type of Participants	Other Visual Field Tests Included
1	Hong et al. [12]	2021	Korea (Seoul)	150(58/92)	62.6(NA)	OAG	24-2 Faster
2	Chakravarti et al. [13]	2021	USA	92(46/46)	66.9 (63.9, 69.9)	OAG, glaucoma suspects	10-2 Standard
3	Phu et al. [18]	2021	Australia (New South Wales)	188 (103/85)	Glaucoma: 65.0 (59.5, 71.0) Glaucoma suspect: 64.0 (57.0, 70.0)	Glaucoma (unspecified), glaucoma suspects	10-2 Fast
4	Phu et al. [11]	2020	Australia (New South Wales)	104 (59/45)	SITA Faster: 62.0 (IQR 52.75, 69.0) SITA Standard: 59.0 (IQR 52.0, 67.8)	Glaucoma (unspecified), glaucoma suspects	24-2 Standard, 24-2 Faster
5	Meshkin et al. [21]	2022	USA	474 (224/250)	64.4 (15.7)	Patients that require visual field testing	Not applicable
6	Behera et al. [22]	2023	India (Puducherry)	60 (32/28)	55.18 (12.19)	Glaucoma (Unspecified)	24-2 Standard, 10-2 Standard
7	Yamane et al. [20]	2021	USA (Columbia)	25 (NA)	54.8 (19.4)	Neuro-ophthalmology patients	10-2 Fast
8	Nishijima et al. [23]	2024	Japan	146 (74/72)	With CVFD (59.5 (57.3–61.6)) Without CVFD (63.2 (55.6–70.8))	POAG, NTG	24-2 Standard, 10-2 Standard

Legend: NA = not available; USA = United States of America; OAG = open-angle glaucoma; POAG = primary open-angle glaucoma; NTG = normal tension glaucoma.

**Table 2:** Comparison across visual field grids and modes, versus the HVF 24-2C Faster.

	Test Grids	24-2C Faster	24-2 Standard	24-2 Faster	10-2 Standard	10-2 Fast
Outcomes						
**Overall VFD detection**			↔ [22,23]	Not compared	Not compared	↔ [20]
**CVFD detection**			− [11,22,23]	− [11]	+ [13,22]	+ [18]
**Visual field indices (MD, PSD, and CMD)**			↔ [11,22]	↔ [11]	↔ [22]	↔ [18]
**Structure–function concordance**			Not compared	− [11,12]	Not compared	+ [18]
**Test duration (speed)**			− [11,22,23]	+ [11]	− [22,23]	− [20]
**Reliability (false +/− rates)**			+ [22]	Not compared	+ [13]	+ [20]

Legend: MD = mean deviation; PSD = pattern standard deviation; CMD = central mean density; green, “+” = superior; yellow, “↔” = similar; red, “-” = inferior.

**Table 3:** Summary of findings—24-2C Faster versus 24-2 Standard.

Outcome	Studies (*n* = Number of Participants)	Effect Estimate
Ability to detect CVFD: TD/TP	3 studies (n = 326)	5.5 more defective points in 24-2C Faster (17.5 vs. 12, *p* < 0.001) (n = 60) More central upper CVFDs detected in 24-2C Faster [AUC = 0.85, 95% CI = (0.78, 0.91) vs. AUC = 0.75 95% CI = (0.67, 0.83)], no difference in central lower CVFDs (*p* > 0.05) (n = 162) Tendency to identify more CVFD clusters but not statistically significant across all criteria (*p* > 0.05) (n = 104)
Ability to detect CVFD: PD	3 studies (n = 326)	2 more defective points in 24-2C Faster (6 vs. 4, *p* < 0.001) (n = 60) More central lower CVFDs detected in 24-2C Faster [AUC = 0.81, 95% CI = (0.72, 0.90) vs. AUC = 0.64, 95% CI = (0.53, 0.75), *p* < 0.05], no difference in central upper CVFDs (*p* > 0.05) (n = 162)
Ability to detect peripheral VFD	1 study (n = 60)	No significant difference (*p* > 0.05) (n = 60)
Conventional visual field indices: MD	2 studies (n = 164)	0.891 dB [95% LOA = (−5.125, 3.344)] lower in 24-2 Standard, no systemic difference (ICC = 0.95, 95% CI 0.92–0.97, *p* < 0.001) (n = 60) 0.73 dB, [96.2% CI =(−1.01, −0.06), *p* = 0.0038] lower in 24-2C Faster (n = 104)
Conventional visual field indices: PSD	2 studies (n = 164)	0.694 dB [95% LOA = (−2.409, 3.797)] higher in 24-2 Standard, no systemic difference (ICC = 0.93, 95% CI 0.89–0.96, *p* < 0.001) (n = 60) No significant difference (*p* > 0.05) (n = 104)
Conventional visual field indices: CMD	1 study (n = 104)	0.35 dB [96.2% CI = (−0.70, −0.03), *p* = 0.0226] lower in 24-2C Faster (n = 104)
Macular S-F concordance	1 study (n = 104)	Higher ratio of identified functional defects compared to structural defects in 24-2C Faster (*p* < 0.0001) (n = 104)
Time taken	3 studies (n = 326)	Faster in 24-2C Faster: 155.0 s, IQR = (140.0–174.0 s) vs. 314.0 s, IQR = (283.8–338.0 s), *p* < 0.001, (n = 104) 210.5 s, IQR = (176.25–247.25 s) vs. 408 s, IQR = (338.75–471.5 s), *p* < 0.001, (n = 60) 165.0 s, SD = (161–170 s) vs. 308.0 s, SD = (302–315 s), *p* < 0.001, (n = 162)
Sensitivity/false negatives	1 study (n = 60)	Lower in 24-2 Standard [4.5%, IQR = (0–7%) vs. 7.5%, IQR = (0.0–12.75%), *p* < 0.01] (n = 60)
Specificity/false positives	1 study (n = 60)	Lower in 24-2C Faster but not significant [0%, IQR = (0–4%) vs. 1%, IQR = (0.0–2.8%), *p* = 0.34] (n = 60)

Legend: TD = total deviation; TP = total probability.

**Table 4:** Summary of findings—24-2C Faster versus 24-2 Faster.

Outcome	Studies (n = Number of Participants)	Effect Estimate
Ability to detect CVFD	1 study (n = 104)	2 more clusters of CVFD out of 64 (3.1%) identified by 24-2C Faster, tendency to identify more CVFD clusters but not statistically significant across all criteria (*p* > 0.05) (n = 104)
Ability to detect overall VFD	-	Not reported
Conventional visual field indices: MD	1 study (n = 104)	0.02 dB [96.7% CI = (−0.33, −0.30 dB), *p* = 0.9715] lower in 24-2C Faster (n = 104)
Conventional visual field indices: PSD	-	Not reported
Conventional visual field indices: CMD	1 study (n = 104)	0.13 dB [96.7% CI = (0.27, 0.04), *p* = 0.2769] lower in 24-2 Faster (n = 104)
Macular S-F concordance	2 studies (n = 336)	Greater in average, hemimacular, superotemporal, inferotemporal parafoveal sectors in 24-2 Faster (*p* < 0.05), similar in superocentral, superonasal, inferocentral, inferonasal parafoveal sectors (*p* > 0.10) (n = 232) More significant defects at regions with structural loss in 24-2C Faster at *p* < 0.05 and *p* < 0.01, higher ratio of identified functional defects compared to structural defects in 24-2C Faster (*p* < 0.0001) (n = 104)
Time taken	1 study (n = 162)	Faster in 24-2 Faster: 125.5 s, IQR = (110.5–148.8 s) vs. 154.5 s, IQR = (136.3–181.8 s); median difference = 26.0 s, *p*< 0.0001 (n = 162)
Sensitivity/false negatives	-	Not reported
Specificity/false positives	-	Not reported

**Table 5:** Summary of findings—24-2C Faster versus 10-2 Standard.

Outcome	Studies (n = Number of Participants)	Effect Estimate
Ability to detect CVFD: TD	1 study (n = 165)	Moderate-to-substantial agreement between 10-2 Standard and 24-2C Faster (ĸ = 0.477–0.708) (n = 165)
Ability to detect CVFD: PD	1 study (n = 165)	Moderate-to-substantial agreement between 10-2 Standard and 24-2C Faster (ĸ = 0.397–0.689) (n = 165)
Ability to detect overall VFD	-	Not reported
Conventional visual field indices: MD	1 study (n = 60)	1.635 dB [95% LOA = (−9.124 to 5.854)] lower in 24-2C Faster, no systematic difference [ICC = 0.80, 95% CI = (0.687, 0.876), *p* < 0.001] (n = 60)
Conventional visual field indices: PSD	1 study (n = 60)	0.776 dB [95% LOA = (−4.678 to 6.230)] higher in 24-2C Faster, no systematic difference [ICC = 0.80, 95% CI = (0.690–0.877), *p* < 0.001] (n = 60)
Conventional visual field indices: CMD	-	Not reported
Macular S-F concordance	-	Not reported
Time taken	2 studies (n = 222)	Faster in 24-2C Faster: 165.0 s, SD = (161–170 s) vs. 343 s, SD = (334–353 s), *p* < 0.001 (n = 162) Median = 210.5 s vs. 244.5 s (n = 60)
Sensitivity/false negatives	1 study (n = 165)	Higher sensitivity in 10-2 Standard (1.0 vs. 0.97) (n = 165)
Specificity/false positives	1 study (n = 165)	Higher specificity in 10-2 Standard (1.0 vs. 0.94) (n = 165)

**Table 6:** Summary of findings—24-2C Faster versus 10-2 Fast.

Outcome	Studies (n = Number of Participants)	Effect Estimate
Ability to detect CVFD: TD	3 studies (n = 267)	2.6 times more defective points identified by 24-2C Faster (46 vs. 17.5) (n = 60) More CVFD identified by 24-2C Faster (58 (43.9%) vs. 42 (31.8%)) (n = 165) Similar between 24-2C Faster and 10-2 Fast at *p* < 0.05 (24-2C vs. 10-2; 4.16 (0.71) vs. 4.28(0.73), *p* = 0.767), *p* < 0.02 (24-2 vs. 10-2; 3.15 (0.69) vs. 3.28 (0.69), *p* = 0.791) and *p* < 0.01 significance (24-2C vs. 10-2; 2.45 (0.63) vs. 2.58 (0.63), *p* = 0.651) (n = 42)
Ability to detect CVFD: PD	3 studies (n = 267)	2.8 times more defective points identified by 24-2C Faster (17 vs. 6) (n = 60) More CVFD identified by 24-2C Faster (56 (42.4%) vs. 42 (31.8%)) (n = 165) Similar between 24-2C Faster and 10-2 Fast at *p* < 0.05, *p* < 0.02, *p* < 0.01 levels (*p* > 0.70) (n = 42)
Ability to detect CVFD: Others	1 study (n = 188)	More central locations with a visual field defect detected by 10-2 Fast (*p* = 0.02), more CVFDs for all contiguity conditions identified by 10-2 Fast (2+ to 6+; *p* < 0.0001) (n = 188)
Ability to detect overall VFD	-	Not reported
Conventional visual field indices: MD	1 study (n = 188)	Similar between 24-2C Faster and 10-2 Fast: mean bias = 0.70 dB, 95% LOA = (−3.8 to 5.2 dB) (n = 188)
Conventional visual field indices: PSD	1 study (n = 188)	Similar between 24-2C Faster and 10-2 Fast: mean bias = 0.92 dB, 95% LOA = (−5.0 to 3.2 dB) (n = 188)
Conventional visual field indices: CMD	-	Not reported
Macular S-F concordance	1 study (n = 188)	Higher S-F concordance in 10-2 Fast (*p* < 0.0001) (n = 188)
Time taken	1 study (n = 42)	Faster in 24-2C Faster: 3:09 vs. 3:58 min, *p* < 0.001 (n = 42)
Sensitivity/false negatives	1 study (n = 42)	Lower false-negative rate in 10-2 Fast 1.15% vs. 3.89% *p* = 0.002 (n = 42)
Specificity/false positives	1 study (n = 42)	Lower false-positive rate in 10-2 Fast (1.09% vs. 2.57%, *p* = 0.043) (n = 42)

## Data Availability

No new data were created or analyzed in this study. Data sharing is not applicable to this article.

## Supplementary Materials

The following supporting information can be downloaded at https://www.mdpi.com/article/10.3390/bioengineering12070711/s1, S1: PRISMA Checklist; S2: Detailed search strategy; S3: Joanna Briggs Institute (JBI) Critical Appraisal Checklist for Cross-Sectional Studies.
