# Supplementary material for: Advancements in Visual Field Testing: A Systematic Review of the 24-2C Test Grid
Source: Bioengineering (Basel). 2025 Jun 29;12(7):711. doi: 10.3390/bioengineering12070711 (PMC12292377; doi:10.3390/bioengineering12070711)
Supplement: Supplementary file 1 [file bioengineering-12-00711-s001.zip › S2+S3_Advancements in Visual Field Testing - A Systematic Review of the 24-2C Test Grid_Eric 0804.pdf]

S2: Detailed Search Strategy

Database	Search Terms
PubMED	("Ocular Hypertension"[Mesh] OR "Glaucoma"[Mesh] OR "glaucoma") AND "24-2c"
EMBASE	('perimetry'/exp OR 'visual field') AND ('glaucoma'/exp OR 'glaucoma') AND '24-2c'
Cochrane Library	("Ocular Hypertension"[Mesh] OR "Glaucoma"[Mesh] OR "glaucoma") AND "24-2c"

S3: Joanna Briggs Institute (JBI) Critical Appraisal Checklist for Cross-Sectional Studies

Author	Year	D1	D2	D3	D4	D5	D6	D7	D8	Total Score	Overall Risk of Bias
Nishijima et al	2024	Yes	Yes	Yes	Yes	Yes	Yes	Yes	Yes	8	Low
Behera et al	2023	Yes	Yes	Yes	Yes	Yes	Yes	Yes	Yes	8	Low
Meshkin et al	2022	Yes	Yes	Yes	Yes	Yes	Yes	Unclear	Yes	7.5	Low
Hong et al	2021	Yes	Yes	Yes	Yes	Yes	Yes	Yes	Yes	8	Low
Chakravarti et al	2021	Yes	Yes	Yes	Yes	Yes	Yes	Yes	Yes	8	Low
Phu et al	2021	Yes	Yes	Yes	Yes	Yes	Yes	Yes	Yes	8	Low
Yamane et al	2021	Yes	Unclear	Yes	Yes	Yes	Yes	Unclear	Yes	7	Low
Phu et al	2020	Yes	Yes	Yes	Yes	Yes	Yes	Yes	Yes	8	Low

D=Domain, D1=Clear inclusion criteria, D2=Detailed description of study subjects and setting, D3=Valid and reliable measurement of exposure, D4=Objective and standard criteria for measurements of condition, D5=Identification of confounding factors, D6=Strategies to deal with confounding factors, D7=Valid and reliable measurement of outcomes, D8= Appropriate statistical analysis
